# Supplementary material for: Coherent photoelectrical readout of single spins in silicon carbide at room temperature
Source: Nat Commun. 2025 Apr 15;16:3405. doi: 10.1038/s41467-025-58629-1 (PMC12000510; doi:10.1038/s41467-025-58629-1)
Supplement: Supplementary file 1 — Supplementary Information [file 41467_2025_58629_MOESM1_ESM.pdf]

Supplementary Information for
Coherent photoelectrical readout of single spins in silicon carbide at room temperature

Tetsuri Nishikawa[1,*], Naoya Morioka[1,2,*,†], Hiroshi Abe[3], Koichi Murata[4], Kazuki Okajima[1], Takeshi Ohshima[3,5], Hidekazu Tsuchida[4], and Norikazu Mizuochi[1,2,6,†]

1. Institute for Chemical Research, Kyoto University, Uji 611-0011, Japan

2. Center for Spintronics Research Network, Institute for Chemical Research, Kyoto University, Uji 611-0011, Japan

3. National Institutes for Quantum Science and Technology, Takasaki 370-1292, Japan

4. Central Research Institute of Electric Power Industry, Yokosuka 240-0196, Japan

5. Department of Materials Science, Tohoku University, Sendai 980-8579, Japan

6. International Center for Quantum-field Measurement Systems for Studies of the Universe and Particles (QUP), KEK, Tsukuba 305-0801, Japan

* These authors contributed equally to this work.

[†]e-mail: morioka.naoya.8j@kyoto-u.ac.jp, mizuochi@scl.kyoto-u.ac.jp

Supplementary Note 1: Device design and characterisation

As discussed in the main text, we use a back-to-back Schottky photocurrent detector that operates above the flat-band condition. We design the electrode gap L such that the device has a flatband voltage[1] $V_{\mathrm{FB}} = eN_{\mathrm{A}}L^2/2\varepsilon_{\mathrm{S}}$ at around 10 V. The measured voltage dependence of the photocurrent from a single defect and the background are shown in Supplementary Fig. 1a. The photocurrent saturates at an applied voltage of 10 V, which agrees with the expected flatband voltage of 9.1 V considering the net acceptor density $N_{\mathrm{A}} = 2 \times 10^{14}$ cm^{-3} and $L = 7.1$ μm. The DC dark current is characterised and shown in Supplementary Fig. 1b. The dark current at 10 V is about 10 fA, which is sufficiently low to detect fA scale small spin signal. Based on these analyses, we perform all experiments in the main text at 10 V. We note that the dark current observed in Supplementary Fig. 1b may not solely reflect the Au/SiC contact's properties because the dark current contains the parasitic leakage current of SiO_2 under the bonding pad whose area is approximately 200 times larger than the Schottky contact. Also, it may contain the leakage current on the SiC surface.

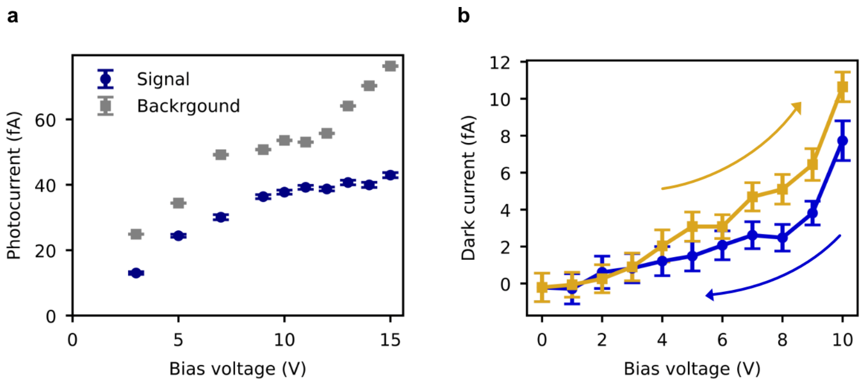

Supplementary Figure 1: *I*-*V* characteristics of the fabricated device.

a Bias voltage dependence of the photocurrent from defect A (blue dots) and from the background (grey squares) measured with lock-in detection by modulating the laser intensity. The error bars represent ±1 standard error. **b** Bias-dependent DC dark current. The blue dots are measured from 10 V to 0 V, and the yellow squares are measured from 0 V to 10 V, as indicated with arrows. The error bars show the standard deviation.

Supplementary Note 2: Photoelectrical measurement with 730 nm excitation

Supplementary Figure 2a shows the photocurrent image obtained with the 730 nm laser used for the optical characterisation. The scan region is the same as for Fig. 2a. For technical reasons, we use the 10^{11} V/A gain transimpedance amplifier for this measurement. It is unable to identify single-defect-like features in the figure. We observe more than one order of magnitude larger current with 730 nm than with 905 nm. The laser power dependence of the photocurrent with 730 nm laser exhibited linear and quadratic components as shown in Supplementary Fig. 2b. We assume that the linear component (0.23 pA/mW) originates from the one-photon ionisation of defects such as carbon vacancies, and the quadratic component (6.1×10^{-3} pA/mW^2) can be attributed to the two-photon band-to-band electron-hole pair generation because the laser photon energy (1.70 eV) is higher than half of the bandgap (3.24 eV). The two-photon absorption coefficient at 730 nm was reported to be $\beta_{2\mathrm{PA}} = 2.2\times10^{-13}$ m/W in semi-insulating 4H-SiC[2] and 2.0×10^{-13} m/W by theoretical calculation[3]. Assuming one electron-hole pair is generated by absorbing two photons (the ideal quantum efficiency), the electron-hole pair generation rate per unit volume is $G_{2\mathrm{PA}} = \beta_{2\mathrm{PA}}\lambda I_\mathrm{p}^2/2hc$ [4], where λ is the excitation laser wavelength, h is the Planck constant, c is the speed of light, and I_p is the power density distribution of the laser in SiC. To simplify the analysis, we ignore the laser power loss due to one- and two-photon absorption and by the free carrier absorption in the laser path in SiC. We approximate the laser power density I_p by a gaussian beam:

$$I_{\mathrm{p}}(\mathrm{r},\mathrm{z}) = \frac{2P}{\pi\{w(z)\}^2}\exp\left(-\frac{2r^2}{\{w(z)\}^2}\right),$$

$$w(z) = w_0\sqrt{1+\left(\frac{z-z_0}{z_{\mathrm{R}}}\right)^2},$$

where r is the distance from the beam center in the xy plane, z is the depth from the surface, $z_0 \approx 1.7$ μm is the focal depth in SiC from the surface, P is the total laser power, w_0 is the beam waist radius, and $z_{\mathrm{R}} = \pi n w_0^2/\lambda$ is the Reyleigh range with n=2.6 as the refractive index of SiC. The theoretical beam waist radius for 730 nm laser with the numerical aperture NA = 1.45 is estimated to be about 0.15 μm. The $1/e^2$ radius of the fluorescence image spot size is 0.24 μm under the saturation power. As the pinhole diameter (=100 μm) is larger than the Airy disk diameter calculated at 950 nm (=80 μm), the diameter of the fluorescence image is limited by either the diffraction limit or the excitation laser diameter. Therefore, the laser diameter is in the range of w_0 =0.15–0.24 μm, and the corresponding z_{R} = 0.25–0.64 μm. The photocurrent by two-photon absorption can be calculated by $I_{2\mathrm{PA}} = \iiint eG_{2\mathrm{PA}}dV$, where the volume integration is performed in the region where the generated charges are collectable by the electrodes. Since the diameter of the laser spot is much smaller than the distance of the electrode, the Gaussian beam distribution is first integrated in the xy plane to infinity as an approximation. Then, the integration is performed to the depth $z_{\max}$, resulting in

$$I_{2\mathrm{PA}} = \frac{e\beta_{2\mathrm{PA}}\lambda P^2}{2\pi hc}\int_0^{z_{\max}}\frac{dz}{\{w(z)\}^2} = \frac{e\beta_{2\mathrm{PA}}n}{2hc}\left(\arctan\frac{z_0}{z_{\mathrm{R}}} + \arctan\frac{z_{\max}-z_0}{z_{\mathrm{R}}}\right)P^2.$$

Assuming that the charge collection works within the depletion layer width, $z_{\max}$ is approximately 7–8 μm. Considering the values above, we estimate the value $I_{2\mathrm{PA}}/P^2$ to be about 0.6–0.7 pA/mW^2. Although we observe one order of magnitude smaller total photocurrent and two orders of magnitude smaller quadratically dependent photocurrent in the experiment, this analysis suggests that the two-photon absorption may need to be considered at 730 nm.

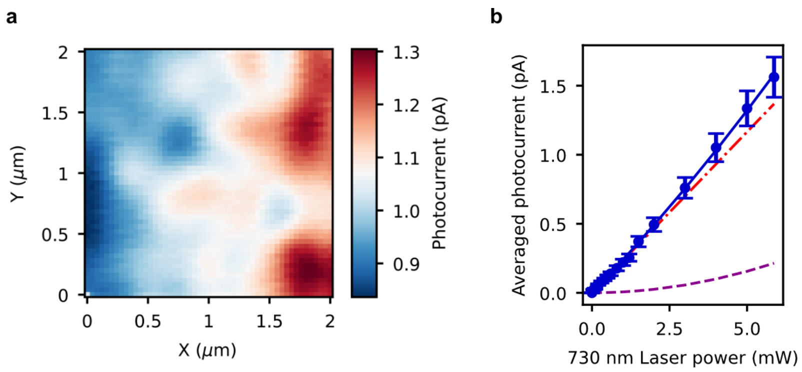

Supplementary Figure 2: **Photocurrent characteristics with 730 nm excitation.**

a Photocurrent scanning image with 730 nm excitation at 4 mW with square-wave amplitude modulation at 13 Hz for lock-in detection. **b** Laser dependence of the photocurrent with 730 nm laser. Blue dots are experimental data with error bars representing ±1 standard error, and the blue solid line is a fit with a quadratic function about a power P, $aP^2 + bP$. The parameters are found to be $a =$ 6.1×10^{-3} $\mathrm{pA/mW^2}$ and $b = 0.23$ $\mathrm{pA/mW}$. The red dash-dot line and the purple dashed line show the linear and quadratic components of the fitting, respectively.

Supplementary Note 3: Comparison of Rabi oscillation between ODMR and PDMR

As shown in Figs. 3d and 3e, we observe the Rabi oscillation of a single V2 centre by PDMR. Here we compare the Rabi oscillation measured by PDMR and ODMR. Supplementary Fig. 3 shows the Rabi signals of a single V2 centre (defect A) by ODMR and PDMR at the same RF and magnetic field conditions. Here, the PDMR is performed with the two-step laser modulation by the AOM with a 150 ns pulse at 20 mW followed by an 850 ns initialisation pulse at 1.5 mW, and both the ODMR and PDMR signals are processed with the background subtraction described in Methods. We observe Rabi oscillations with the frequencies of 4.12 ± 0.03 and $4.11 \pm$ 0.07 MHz in ODMR and PDMR, respectively, and they are in good agreement within the margin of error. Therefore, we confirm that PDMR and ODMR observe the identical spin manipulation signal.

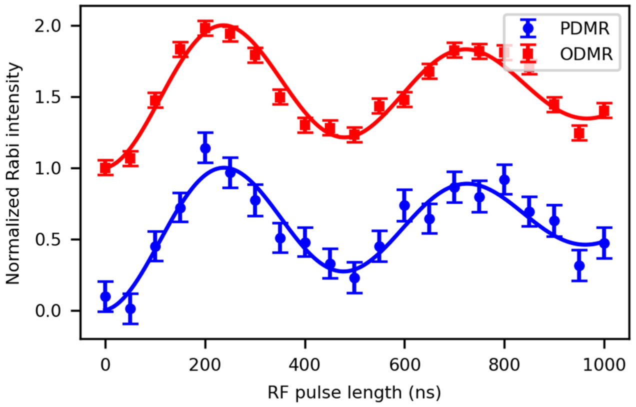

Supplementary Figure 3: Rabi oscillations measured with ODMR and PDMR.

Symbols represent the experimental data, and the solid lines show the fit with a damped cosine function. The error bars in this figure show ±1 standard error.

Supplementary Note 4: Optimisation of signal-to-noise ratio in laser-power-dependent ODMR

As shown in Fig. 2c, we observe 9.9 kcps as the saturation count rate of a single V2 centre at defect A, comparable to those in previous studies characterised with confocal microscopy using oil immersion objective lenses[11,12], indicating the system's comparable photon detection efficiency to previous studies. We point out that we do not observe any blinking or bleaching effects for defects A and C, although our material is p-type, indicating the stability of the negative charge state for these defects. As we characterise defects near the surface, downward surface band bending may stabilise the charge state. The observed pulsed ODMR contrast (4% for defects A and C) is also similar to previously reported ODMR contrasts (2% for Rabi oscillation[5], 4%–6% for continuous-wave ODMR[5,6]). Therefore, the optical system and defects used in this study are suited to compare the SNR performance of ODMR and PDMR.

As our target is a coherent spin-state readout, we use a pulsed ODMR sequence rather than a continuous-wave measurement. PDMR shares the same total sequence length, timing of the laser and RF pulses, and strength of the RF pulse as ODMR, as depicted in Fig. 2e, except for the laser pulse shape and length. The laser pulse is 1 μs long with varying power. The rise time of the AOM is 12.5 ns. The interval between the end of the laser pulse and the RF pulse is $T_{\mathrm{wait}} = 1000$ ns for the defect to allow enough time to decay to the ground state. The total sequence length is $T_{\mathrm{seq}} = 2530$ ns.

The spin signal in optical detection appears only in the beginning of the time window. Therefore, we record the detected photon time histogram during the laser pulse, and then the integration time window

is optimised to obtain the best shot-noise limited SNR in ODMR by post-processing for each laser power condition. The shot-noise limited SNR is calculated using data accumulated for 150 s for each data point. The obtained time-gating conditions are used to calculate the SNR with data accumulated for 30 s per point displayed in Fig. 4c. We obtain the best shot-noise limited SNR at a laser power of 1.0 and 0.8 mW for defects A and C, respectively. We observe almost shot-noise-limited SNR for ODMR.

Supplementary Note 5: Wide-area imaging between the electrodes and single-defect-like photocurrent features with absence of photoluminescence

Supplementary Figures 4a and 4b show the optical (excitation wavelength: λ_{exc}=730 nm, detection wavelength: λ_{det}>900 nm) and photocurrent (λ_{exc}=905 nm) image scanned in the XY plane (parallel to the sample surface) in a wide area between the electrodes, respectively. Many spots including defects A, B and C characterised in the main text are seen in the figures. In addition, there are several single-defect-like features in the photocurrent image which do not appear in the optical image. One example is the photocurrent spot X next to defect A shown in Fig. 2d and Supplementary Fig. 4b, which is not visible in Fig. 2a and Supplementary Fig. 4a. The photoluminescence of spot X is absent with another long-pass filter (λ_{det}>830 nm) and with two excitation wavelengths (λ_{exc}=730 nm and 671 nm: shown in Supplementary Figs. 4c and 4d, respectively). Supplementary Figure 4e. shows the cross-sectional X–Z plane photocurrent image of defect A and spot X (λ_{exc}=905 nm) measured at Y=1 μm of Fig. 2d. Spot X is about 850 nm deeper than defect A (single V2 centre), and defect A and spot X have similar size in XYZ directions with a comparable photocurrent intensity. Therefore, spot X is considered a single defect that can generate photocurrent by a two-step ionization at 905 nm, a process similar to the V2 centre. The photoluminescence of spot X, if present, may appear above 1060 nm, the spectral sensitivity limit of the silicon avalanche photodiode used in this study.

Defect C is identified to be a single defect by an optical autocorrelation function measurement, as shown in Supplementary Fig. 4f. The saturation photon count rate is 6.6 kcps, which is not much different from defect A. From the PDMR spectra shown in Supplementary Fig. 5, we find that the resonance frequency without the applied magnetic field is 74.5 ± 0.1 MHz, and the splitting of the resonance frequencies of the two transitions under the magnetic field aligned to the V2 centre's axis is 148.3 ± 0.3 MHz. These values are slightly larger than the V2 centre ($2D$ and $4D$, respectively, with D=35.0 MHz). Considering the similar fluorescence and spin properties, defect C is possibly a modified silicon vacancy defect, a silicon vacancy with a nearby carbon antisite[7]. To precisely

determine the zero-field splitting parameters D and E of defect C, magnetic field angle and strength dependence of the resonance frequency is necessary. However, from the resonance frequencies in Supplementary Fig. 5 and assuming the same S_z axis direction as the V2 centre, we estimate $D \approx 37.1$ MHz and $E \approx 2$ MHz.

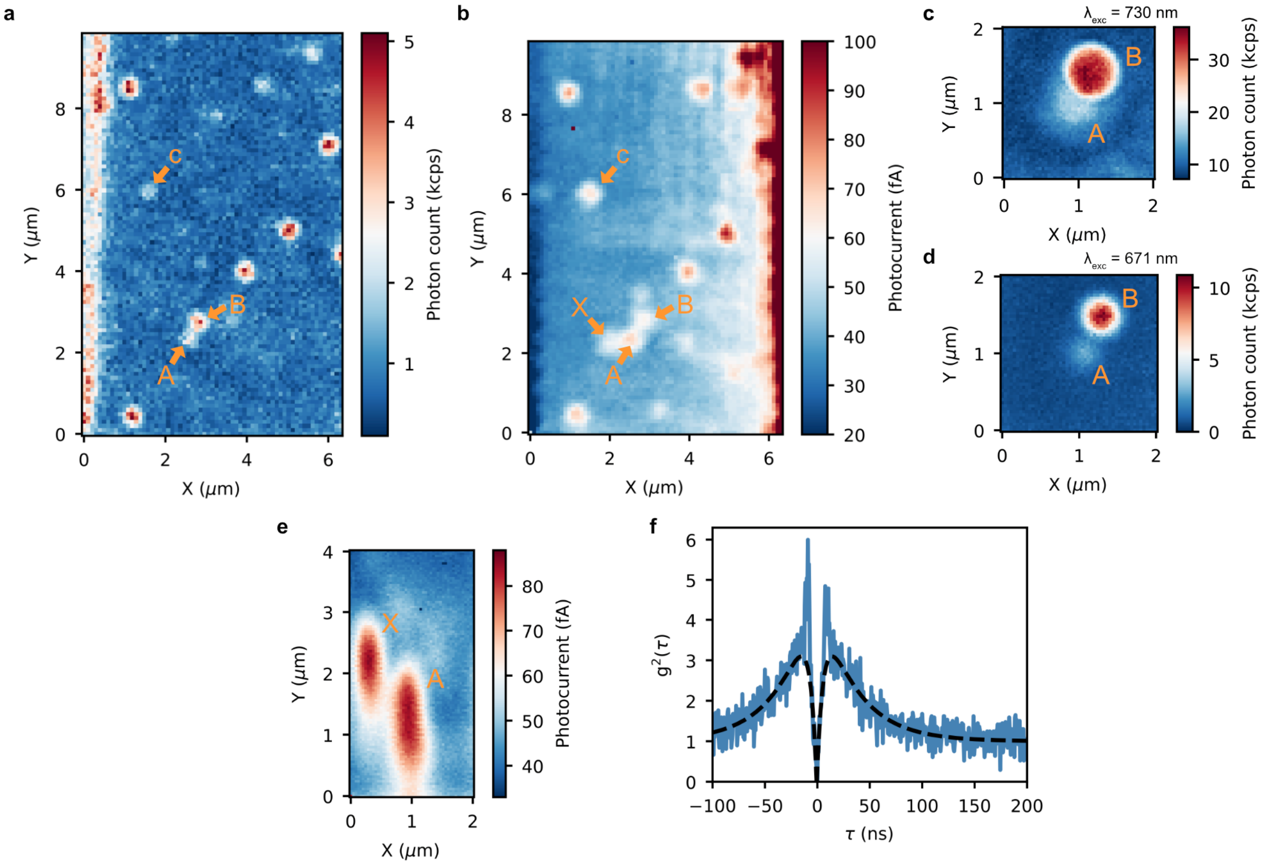

Supplementary Figure 4: **Supplemental photoelectrical and fluorescence images in the PDMR device and autocorrelation function of defect C.**

a Wide-range fluorescence image between the electrodes. Labelled orange arrows point to defects A–C. **b** Photocurrent image in the same area measured at 4 mW. **c** Fluorescence image with 730 nm laser excitation at 2 mW with the detection wavelength range of λ_{det}>830 nm. **d** Fluorescence image with 671 nm laser excitation at 100 μW with λ_{det}>830 nm. c and d are measured in the same area as Fig. 2a. **e** X-Z plane photocurrent image at Y=1 μm of Fig. 2d in the main text. The laser power is 4 mW. **f** Optical autocorrelation function data of defect C after the background correction (solid line) and the fit curve (dashed line). Note: Only the autocorrelation data in **f** was measured using a different confocal microscope setup, with an excitation laser wavelength of 789 nm and an objective lens with NA = 1.35.

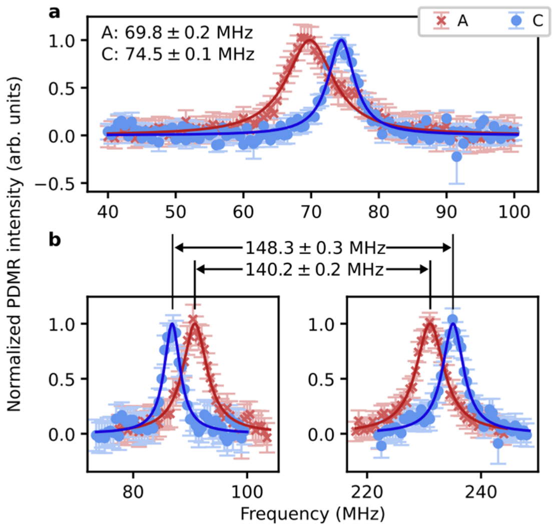

Supplementary Figure 5: Comparison of PDMR spectra of single defects A and C.

a PDMR spectra without an applied magnetic field. The inset text shows the resonance frequency from the fitting to Lorentzian curves (solid lines). **b** PDMR spectra under applied magnetic field aligned to the *c*-axis of the sample. The error bars in this figure show ±1 standard error.

Supplementary Note 6: Photocurrent generation dynamics of V2 centre

The PDMR mechanism for a V2 centre has been considered similar to that of an NV centre in diamond[8,9], which is based on the photo-induced charge-state transition[10] between the single negative and the neutral states[8]. However, the details for the ionisation and recharging processes of the silicon vacancy are unrevealed because the neutral silicon vacancy has not been observed optically, unlike the NV centre in diamond. Here, we estimate the ionisation dynamics of a single V2 centre based on a rate model and the experimental laser power dependence of the photocurrent. We use a four-level model considered in diamond NV centres[10], including a ground state (GS), an excited state (ES), a metastable state (MS) of the V2 centre (single negative state) and the neutral state (V_{Si}^0), as depicted in the main text Fig. 1a.

The rate equations for these four states are written as

$$\dot{n}_{\mathrm{GS}} = -\gamma_{\mathrm{e}} P n_{\mathrm{GS}} + k_1 n_{\mathrm{ES}} + k_3 n_{\mathrm{MS}} + \gamma_{\mathrm{r}} P n_{\mathrm{V_{Si}^0}}, \quad (1)$$

$$\dot{n}_{\mathrm{ES}} = \gamma_{\mathrm{e}} P n_{\mathrm{GS}} - (k_1 + k_2 + \gamma_{\mathrm{i}} P) n_{\mathrm{ES}}, \quad (2)$$

$$\dot{n}_{\mathrm{MS}} = k_2 n_{\mathrm{ES}} - k_3 n_{\mathrm{MS}}, \quad (3)$$

$$\dot{n}_{\mathrm{V_{Si}^0}} = \gamma_{\mathrm{i}} P n_{\mathrm{ES}} - \gamma_{\mathrm{r}} P n_{\mathrm{V_{Si}^0}}, \quad (4)$$

where n_i (i = GS, ES, MS, V_{Si}^0) is the population of each state satisfying $\sum_i n_i = 1$. k_1, k_2, and k_3 are the decay rates for ES→GS, ES→MS, and MS→GS, respectively, and γ_{e}, γ_{i}, and γ_{r} are the rates of the excitation (GS→ES), the ionisation (ES→V_{Si}^0), and the recharging (V_{Si}^0→GS) per unit laser power, respectively. P is the laser power. The steady-state photocurrent I is calculated from the steady-state

excited state population $n_{\mathrm{ES}}(\infty)$ as $I = e\eta_{\mathrm{c}}\gamma_{\mathrm{i}}Pn_{\mathrm{ES}}(\infty)$, where η_{c} is the carrier collection efficiency, and e is the elementary charge. The solution is

$$I = \frac{\alpha P^2}{1 + P/P_0}, \tag{5}$$

where

$$\alpha = e\eta_{\mathrm{c}}\gamma_{\mathrm{i}}\gamma_{\mathrm{e}}\,\tau_{\mathrm{ES}}, \tag{6}$$

$$P_0^{-1} = \gamma_{\mathrm{e}}\tau_{\mathrm{ES}}\left(1 + \frac{k_2}{k_3} + \frac{\gamma_{\mathrm{i}}}{\gamma_{\mathrm{r}}} + \frac{\gamma_{\mathrm{i}}}{\gamma_{\mathrm{e}}}\right). \tag{7}$$

Here, $\tau_{\mathrm{ES}} = 1/(k_1 + k_2)$ is the excited-state lifetime. P_0 corresponds to the saturation power of the transition from GS to ES. The power dependence of the photocurrent changes from quadratic ($P < P_0$: low-power regime) to linear ($P > P_0$: high-power regime). P_0 is also recognised as the half saturation power of the fluorescence. α is the slope of the photocurrent against the excitation laser power in the high-power regime. Here, we can use the measured α and P_0 for defect A described in the main text to infer the excitation and ionisation rates. In calculation, we need to convert the fundamental frequency component $I_{\mathrm{ph,LIA}}$ measured with the lock-in amplifier to the amplitude of the original square waveform, $\pi I_{\mathrm{ph,LIA}}/\sqrt{2}$, and therefore we use the $\alpha' = \pi\alpha/\sqrt{2}$ as the conveted parameter in the calculation below. To calculate the excitation and ionisation rates, we introduce an approximation of $\gamma_{\mathrm{i}}/\gamma_{\mathrm{r}} \ll 1$. This approximation is reasonable based on the photostable characteristics of single V2 centres without blinking under photoexcitation[5,11]; V2 can be quickly converted to the negative state after photoionisation to the neutral state. Under this approximation and the observed small P_0 indicating $\gamma_{\mathrm{i}}/\gamma_{\mathrm{e}} < 1$, we find from Eqs. (6) and (7) that

$$\gamma_{\mathrm{e}} \approx \left\{\tau_{\mathrm{ES}}P_0\left(1 + \frac{k_2}{k_3}\right)\right\}^{-1} - \frac{\alpha' P_0}{e} \approx \left\{\tau_{\mathrm{ES}}P_0\left(1 + \frac{k_2}{k_3}\right)\right\}^{-1}, \tag{8}$$

$$\gamma_{\mathrm{i}} \approx \frac{\alpha' P_0}{e\eta_{\mathrm{c}}}\left(1 + \frac{k_2}{k_3}\right), \tag{9}$$

$$\frac{\gamma_{\mathrm{i}}}{\gamma_{\mathrm{e}}} = \frac{\alpha'\tau_{\mathrm{ES}}P_0^2}{e\eta_{\mathrm{c}}}\left(1 + \frac{k_2}{k_3}\right)^2. \tag{10}$$

Here, in the case of the V2 centre, the second term in Eq (8) is negligible compared to the first term. Using the measured α and P_0 in this study and the rates (k_1, k_2, and k_3) at room temperature reported by Fuchs et al.[11] and Singh et al.[6], the excitation and ionisation rates are calculated as Supplementary Table 2. The excited-state lifetime τ_{ES} used in the calculation is also obtained from the same references. The independent measurement of τ_{ES} reports about 6 ns[12] which is consistent with the used rates. From the saturated photocurrent at applied bias voltage discussed in Supplementary Note 1, $\eta_{\mathrm{c}} \approx 1$ is expected. Therefore, the ionization rate is about 50 times slower than the excitation rate at 905 nm. In addition, the absorption cross-section of a V2 centre at 905 nm is calculated to be approximately 0.4×10^{-16} cm^2 given the transmittance of the objective (0.74 for 905 nm), which is one-third of the

reported value for 785 nm excitation[6,11]. Optimisation of the wavelength, therefore, can improve the spin-to-charge conversion efficiency to enhance the PDMR intensity at lower excitation powers.

Supplementary Table 1: Excitation and ionisation rates of the single V2 centre.

Reference of rates	τ_{ES} (ns)	$k_{\mathrm{em}}/k_{\mathrm{mg}}$	γ_{e}^{-1} (ns·mW)	γ_{i}^{-1} (μs·mW)	$\gamma_{\mathrm{i}}/\gamma_{\mathrm{e}}$
Ref. 11, 3 level	5.2	6.4	17	$1.1\eta_{\mathrm{c}}$	$1.6 \times 10^{-2}\eta_{\mathrm{c}}^{-1}$
Ref. 11, 4 level	5.3	7.5	20	$0.9\eta_{c}$	$2.2 \times 10^{-2}\eta_{\mathrm{c}}^{-1}$
Ref. 6, 3 level	7.5	6.0	24	$1.1\eta_{\mathrm{c}}$	$2.1 \times 10^{-2}\eta_{\mathrm{c}}^{-1}$

Supplementary Note 7: Estimation of signal-to-noise ratio at higher ionisation laser power

Due to the small ionisation cross-section and the limited laser power for the ionisation, the observed SNR is possibly limited by the insufficient ionisation efficiency. Therefore, we perform a numerical simulation of PDMR with a short-pulse ionisation laser to estimate the approximate SNR when a higher ionisation laser power is available. For V2 centres in silicon carbide, spin-dependent internal dynamics rates and ground-state spin polarisation after the optical pumping have not been fully determined at room temperature. Therefore, we use a simplified model with several approximations and assumptions.

We show the simulation model in Supplementary Fig. 7a. As the first approximation, we use the rates from ES to GS (k_{eg}) and from ES(±1/2) to MS (k_{em1}) reported in a study at a low temperature by Liu et al.[13], k_{eg}^{-1} =17.8 ns^{-1}, k_{em1}^{-1} =9.2 ns^{-1}. However, the rates from ES(±3/2) to MS (k_{em1}) is varied within the range from 1/35 ns^{-1} to 1/20 ns^{-1}, which includes the value reported by Liu et al. (1/31.2 ns^{-1}) [13] because the room-temperature spin-dependence intersystem crossing rates of V2 centres are unknown. The total intersystem crossing rate can change the ionisation current slightly, but we can roughly estimate the PDMR current. Second, we ignore the transition from the MS to GS during the laser pulse duration of 36 ns, which is shorter than the expected MS lifetime (≈120 ns) [13]. We use this approximation because the spin-dependent rates and the initialised GS spin polarisation are unknown. This approximation may overestimate the spin signal slightly, but we can estimate the approximate signal intensity. Third, we determine the initial GS spin polarisation so that the experimental PDMR contrast 2.0% is reproduced at a laser power of 35 mW, considering the background current (assuming a signal-to-background ratio SBR=1) and the current during the initialisation laser (estimated to be 8.5 fA) for each set of the rates. The ionisation rate and the ratio to the excitation rate are assumed to be

the average of values discussed in Supplementary Note 6, i.e., $\gamma_{\mathrm{i}} \approx 1.0^{-1}$ μs^{-1} mW^{-1} and $\gamma_{\mathrm{i}}/\gamma_{\mathrm{e}} \approx$ 2.0×10^{-2}. The recharging rate γ_{r} from the neutral state is unknown. However, as discussed in Supplementary Note 6, the recharging rate should be much faster than the ionisation rate. Therefore, we assume $\gamma_{\mathrm{r}} = 50\gamma_{\mathrm{i}}$. We also assume that the recharging process does not have a spin preference. In the simulation, we assume a unity carrier collection efficiency $\eta_{\mathrm{c}} = 1$. We numerically solve the rate model and calculate the charge generated during the square laser pulse shape with a length of 36 ns. Then, we add the current from the background and during the initialisation. We consider imperfect π-pulse fidelity (0.79) estimated from the experimental Rabi oscillation. We compare the results with the experimental data obtained using a laser pulse length of 39 ns (Exp.1) and 36.8 ns (Exp.2), which includes rise and fall transitions. The average number of electrons emitted from the defect per laser pulse is calculated in Supplementary Fig. 7b. At the experimental laser power of 35 mW, we obtain about 0.4 electrons per pulse, which agrees with our rough estimation given in the main text. The simulated total current, PDMR spin signal, SNR, and contrast are shown in Supplementary Fig. 7c, d, e, and f, respectively, with corresponding experimental data. For the contrast, we calculate two values: the intrinsic contrast and the value considering the background and initialisation current. Although we determine the GS spin polarisation to reproduce the experimental data only at a laser power of 35 mW, a good agreement between the experimental and simulated contrasts is obtained at other powers. Small differences may come from the ignored power dependence of the SBR. The SNR is calculated as a value for 1-second integration, and we consider the photocurrent shot noise and the system noise $i_{\mathrm{n,sys}}$ as the noise sources. We evaluate the SNR using two different system noises: the total noise observed in the experimental setup ($i_{\mathrm{n,sys}}$=0.40 fA/$\sqrt{\mathrm{Hz}}$) and the amplifier-limited noise ($i_{\mathrm{n,sys}}$=0.24 fA/$\sqrt{\mathrm{Hz}}$). The simulated SNR at $i_{\mathrm{n,sys}}$=0.40 fA/$\sqrt{\mathrm{Hz}}$ is close to the experimental result. However, considering a slightly larger simulated signal intensity in Supplementary Fig. 7d, the system noise may be overestimated. Within this model, the SNR can reach ≈ 5.5 for 1-second integration, roughly 3 times higher than the best experimental SNR=1.7 of PDMR. However, for a more accurate estimation of the SNR limit, knowledge of reliable spin-optical dynamics of the defect at room temperature is necessary, which is out of the scope of this study. Nevertheless, PDMR's potential can be expected from the simulation to achieve an even higher SNR than the value observed in this study, which already exceeds ODMR. Even higher SNR would be expected with better spin-flip fidelity and a smaller background current. For the realisation of a higher SNR for the PDMR of the V2 centre, studying the wavelength dependence of the ionisation cross-section is important to explore better ionisation efficiency.

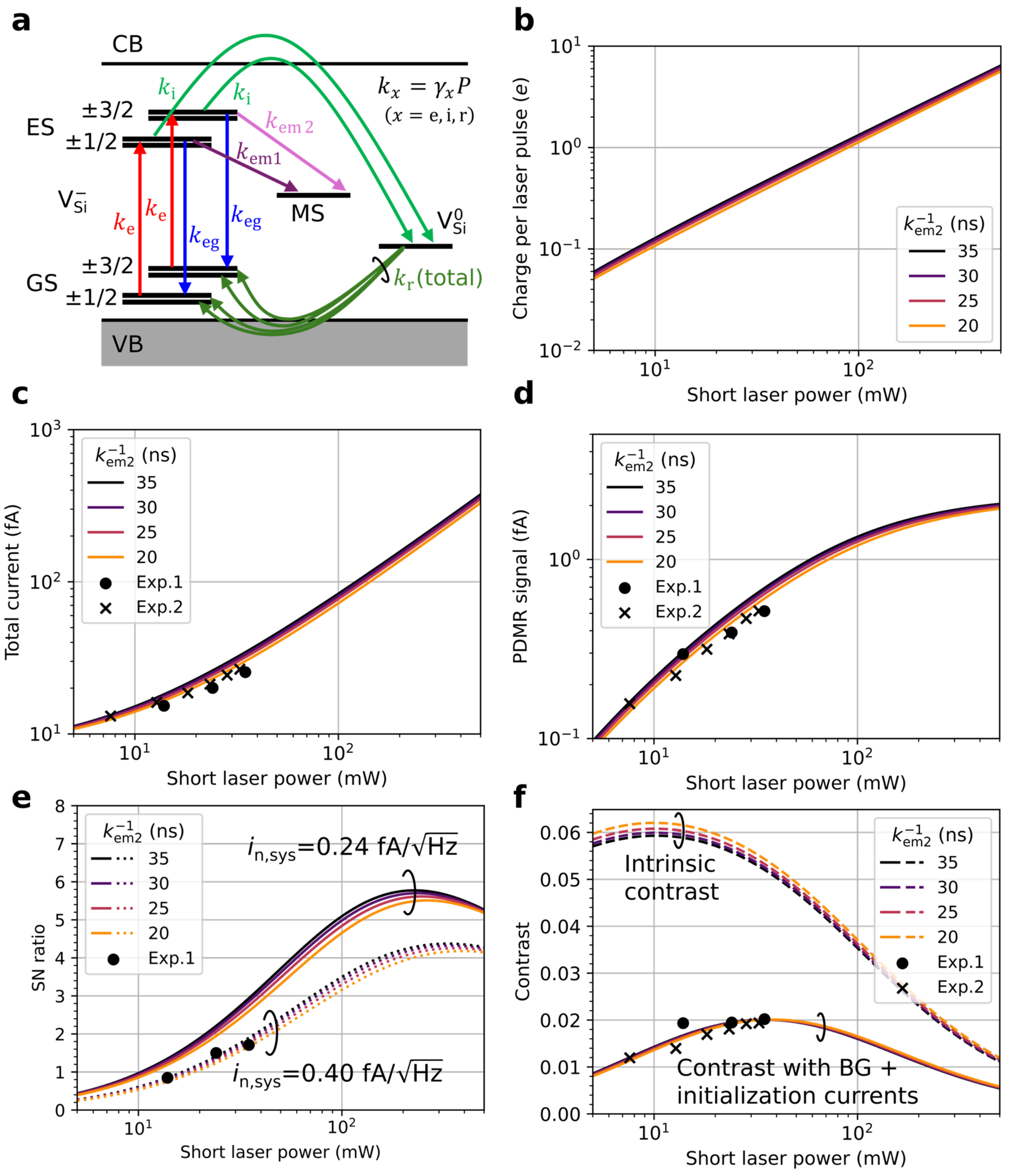

Supplementary Figure 6: **Simulation of the PDMR with a short pulse.**

a Simulation rate model. **b** The number of charges per laser pulse in the unit of elementary charge. **c** The total photocurrent, including the background current and the initialisation current contribution. **d** Spin signal. **e** SNR with different system noise, amplifier limit 0.24 fA/$\sqrt{\text{Hz}}$ and total experimental system noise 0.40 fA/$\sqrt{\text{Hz}}$. **f** Contrast of PDMR with the background and initialisation currents and intrinsic PDMR contrast.

Supplementary References

1. Sze, S. M., Coleman, D. J. & Loya, A. Current transport in metal-semiconductor-metal (MSM) structures. *Solid State Electronics* **14**, 1209–1218 (1971).
2. Guo, X. *et al.* Nonlinear optical properties of 6H-SiC and 4H-SiC in an extensive spectral range. *Opt Mater Express* **11**, 1080 (2021).
3. De Leonardis, F., Soref, R. A. & Passaro, V. M. N. Dispersion of nonresonant third-order nonlinearities in Silicon Carbide. *Sci Rep* **7**, 1–12 (2017).
4. Liu, Y. & Tsang, H. K. Time dependent density of free carriers generated by two photon absorption in silicon waveguides. *Appl Phys Lett* **90**, 211105 (2007).
5. Widmann, M. *et al.* Coherent control of single spins in silicon carbide at room temperature. *Nat Mater* **14**, 164–168 (2015).
6. Singh, H. *et al.* Characterization of single shallow silicon-vacancy centers in 4H−SiC. *Phys Rev B* **107**, 134117 (2023).
7. Davidsson, J. *et al.* Exhaustive characterization of modified Si vacancies in 4H-SiC. *Nanophotonics* **11**, 4565–4580 (2022).
8. Niethammer, M. *et al.* Coherent electrical readout of defect spins in silicon carbide by photo-ionization at ambient conditions. *Nat Commun* **10**, 5569 (2019).
9. Bourgeois, E. *et al.* Photoelectric detection of electron spin resonance of nitrogen-vacancy centres in diamond. *Nat Commun* **6**, 8577 (2015).
10. Aslam, N., Waldherr, G., Neumann, P., Jelezko, F. & Wrachtrup, J. Photo-induced ionization dynamics of the nitrogen vacancy defect in diamond investigated by single-shot charge state detection. *New J Phys* **15**, 013064 (2013).
11. Fuchs, F. *et al.* Engineering near-infrared single-photon emitters with optically active spins in ultrapure silicon carbide. *Nat Commun* **6**, 7578 (2015).
12. Hain, T. C. *et al.* Excitation and recombination dynamics of vacancy-related spin centers in silicon carbide. *J Appl Phys* **115**, 133508 (2014).
13. Liu, D. *et al.* The silicon vacancy centers in SiC: determination of intrinsic spin dynamics for integrated quantum photonics. *npj Quantum Inf* **10**, 72 (2024).
